# Computational methods for optical mapping

**DOI:** 10.1186/2047-217X-3-33

**Published:** 2014-12-30

**Authors:** Lee Mendelowitz, Mihai Pop

**Affiliations:** Center for Bioinformatics and Computational Biology, University of Maryland, College Park, MD USA; Applied Math & Statistics, and Scientific Computation, University of Maryland, College Park, MD USA; Department of Computer Science, University of Maryland, College Park, MD USA

**Keywords:** Optical mapping, Genome mapping, Genome assembly, Structural variation

## Abstract

Optical mapping and newer genome mapping technologies based on nicking enzymes provide low resolution but long-range genomic information. The optical mapping technique has been successfully used for assessing the quality of genome assemblies and for detecting large-scale structural variants and rearrangements that cannot be detected using current paired end sequencing protocols. Here, we review several algorithms and methods for building consensus optical maps and aligning restriction patterns to a reference map, as well as methods for using optical maps with sequence assemblies.

## Introduction

Prior to the advent of cheap high-throughput sequencing technologies and corresponding analytical tools, such as genome assemblers, genomic mapping approaches provided scientists with a first glimpse at the large-scale structure of the chromosomes of organisms. Among the many competing technologies for mapping ( e.g., see [1] for a review of other approaches), the optical mapping technology [2] for the first time, provided the ability to identify the location and order of restriction sites along DNA molecules, thereby enabling the efficient construction of accurate genome-scale restriction maps. Since the initial demonstration of this system in the yeast *Saccharomyces cervisiae*, optical mapping has been used to validate and assist the reconstruction of multiple genomes ranging from bacteria [3] to the human genome [4]. This technology has also been demonstrated to be a powerful tool for comparative genomics allowing the detection of structural variants within genomes [4, 5]. Recently, an evolution of the optical mapping technology – nanocoding – was developed [6], promising higher accuracy and throughput than the original optical mapping system.

**Figure 1:**
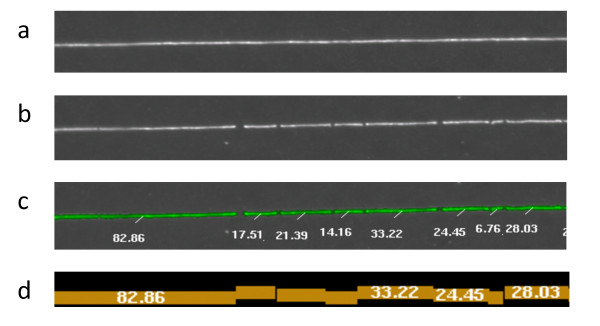
**Optical mapping experiment.** In an optical mapping experiment, stretched DNA molecules are deposited on a charged glass surface using an array of microfluidic channels **(a)** and digested with a methylation-insensitive restriction enzyme that cuts the DNA at specific sequence based recognition sites **(b)**. The stretched DNA relaxes around the cut sites, but in the process, small restriction fragments can be lost through desorption. The DNA molecules are then stained with fluorescent dye and imaged. Restriction fragments are identified with machine vision and the fragment lengths are estimated by integrating fluorescent intensity **(c)**. For each molecule this produces an ordered listing of restriction fragment lengths known as an Rmap **(d)**.

Before describing the computational approaches for analyzing optical (or nanocoding) mapping data, we will briefly describe the key characteristics of these data. The mapping experiment begins with large DNA molecules (hundreds of thousands of base-pairs) which are immobilized on a surface, digested with one or more restriction enzymes, and stained with a fluorescent dye (Figure 1). The series of cuts or nicks produced by the restriction enzyme are detected by imaging the immobilized DNA, and the length between consecutive cut sites is estimated by integrating the fluorescence intensity. The resulting data is an ordered series of fragment lengths, corresponding to the estimation by machine imaging of the distances between nicks or cuts. These data commonly contain a number of errors, such as inaccurate estimates of restriction fragment size (due to non-uniform fluorescent staining), missing or extra restriction sites, or missing small restriction fragments (due to limitations of the experimental and/or imaging components of the system). Furthermore, these data only span individual DNA molecules. Information from multiple overlapping DNA molecules that originate from the same genomic location needs to be combined/assembled in order to construct chromosome-wide maps. The map assembly process can also correct many of the above-mentioned errors. Throughout the following we will refer to single DNA molecule optical maps (the restriction fragments sized and ordered) as *Rmaps* and to the consensus maps of the assembled *Rmap* contigs as *consensus optical maps*.

It should be obvious from this brief description that computational analysis software must be an integral part of the generation and use of optical mapping data. After machine vision software necessary to generate the initial raw data (which is beyond the scope of our review), computational tools are necessary to align to each other and assemble together individual Rmaps, as well as to align the assembled maps to each other (e.g., when identifying structural variants), or to genomic sequences (e.g., to validate or assist the genome assembly process). Below we review the key principles underlying these operations as well as published software tools for using and analyzing optical mapping data.

## Review

### Methods for optical map alignment

**Figure 2:**
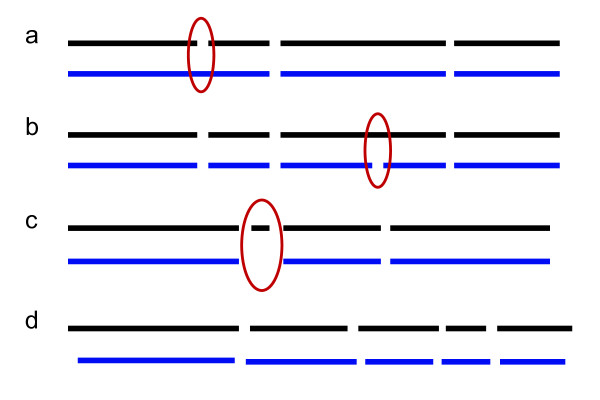
**Optical mapping experimental errors.** Experimental errors in the optical mapping of individual molecules include **(a)** missing enzyme cut sites due to incomplete digestion, **(b)** extra enzyme cut sites due to random breakage of the DNA molecule, **(c)** missing small fragments due to desorption, and **(d)** sizing error due to noise in measurements of fluorescence intensity. The ideal, error-free map is shown in black, and the experimentally observed map is shown in blue.

**Figure 3:**
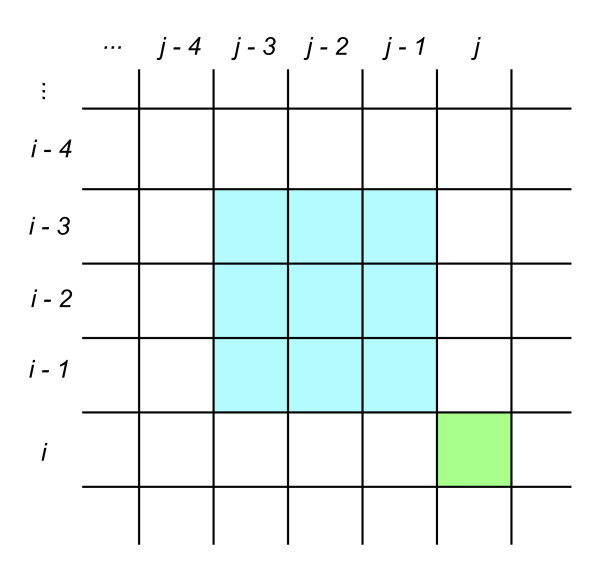
**Dynamic Programming for optical map alignment.** Optical map aligners, such as the aligner by Valouev [7] and SOMA [9] use dynamic programming to compute the optimal scoring alignment. Let cell *(i, j)* in the dynamic programming matrix, colored in green, represent the optimal partial alignment of the query map of *m* fragments through the *i* ^*th*^ restriction site to the reference map of *n* fragments through the *j* ^th^ restriction site such that site *i* is matched to site *j*. To allow for unmatched restriction sites in the alignment, the score for cell *(i, j)* is determined by attempting to extend previously computed alignments in an adjacent *δ* ^2^ region of the matrix, colored in blue. This allows for up to *δ* - 1 consecutive unmatched sites in both the query and the reference. The alignment method is then *O*(*δ* ^2^ *mn*).

One fundamental problem in using genome maps is the task of aligning restriction maps, either to each other or to a genome sequence. The alignment scoring functions must take into account the error characteristics of the mapping experiment, including fragment sizing error, missing and false restriction sites, as well as missing fragments (Figure 2). Dynamic programming algorithms for alignment can accommodate missing restriction sites, false restriction sites, and missing fragments by allowing for different alignment extensions (Figure 3). Alignment methods must accommodate some sizing error since an experimental Rmap fragment size will rarely be an exact match to the corresponding fragment in another Rmap or in the reference genome. For this reason, alignment scoring functions allow for small differences, but penalize large differences in restriction fragment size.

There are several different flavors of the alignment problem: (i) The alignment of individual Rmaps to detect overlaps – a critical step for the *de novo* assembly of an optical consensus map, (ii) the alignment of individual Rmaps to an optical consensus map to call structural variants, or (iii) the alignment of *in silico* restriction maps derived from contigs or scaffolds from sequence assembly to a consensus optical map. Here we review several of the published alignment methods, as well as a method for determining alignment significance.

#### Alignment methods

Valouev *et al.* [7] have developed an alignment algorithm for both finding overlaps between two optical maps and aligning an optical map to a reference map. The scoring function is defined as a log likelihood ratio test for a model that makes the following assumptions: the size of genomic restriction fragments are distributed exponentially; the observations of each restriction site in an optical map are independent Bernoulli processes; the number of false cuts in a given genomic length is a Poisson process; and fragment sizing error is distributed normally with mean zero and variance that scales linearly with the true fragment size. A separate normal sizing error model is used for fragment sizing error for small restriction fragments below a specified threshold. Lastly, the authors put a bound on the number of restriction fragments allowed between consecutively matched restriction sites, leading to a dynamic programming algorithm which runs in time proportional to *mn* where *m* and *n* are the number of restriction sites in the aligned maps (Figure 3). This alignment tool has been successfully used for overlapping Rmaps as part of *de novo* optical map assembly [8].

SOMA [9] is another alignment tool designed specifically for aligning sequence contigs from a genome assembly to a consensus optical map. First, the contigs are converted into an *in silico* restriction map by noting the location of the enzyme’s recognition sites within the contig sequence. Next, the software finds good placements of contigs to the optical map using a dynamic programming algorithm. Lastly, SOMA uses this set of good alignments to select a layout of non-overlapping alignments to the consensus map, in effect constructing a genome-wide scaffold of contigs. The dynamic programming algorithm for alignment uses a chi-squared scoring function to penalize restriction fragment sizing error and a fixed cost penalizing each unaligned site in both the reference map and contig *in silico* map. The statistical significance of alignments is determined by performing a permutation test for each contig with sufficient restriction sites. For contigs with multiple significant alignments, an F-test is used to further filter out secondary alignments by comparing the ratio of the best alignment’s chi-square score to that of each the secondary alignment. Finally, SOMA uses a scheduling algorithm to find non-overlapping placements of the contigs to the optical map. The goal is to find the maximum weight layout, where each contig placement is weighted by the match significance, given as the *p-value* from either the permutation test or the F-test. Several different scheduling algorithms are considered, including a greedy algorithm which prioritizes the placement of contigs with the highest match significance, provided it does not overlap the best scoring scheduling of the remaining fragments (GREEDY); an expensive algorithm which enumerates all possible layouts using depth-first search with pruning of low scoring layouts (ASTAR); and a simple, heuristic approach which places contigs in descending order of match significance such that there are no overlaps (match filtering).

TWIN [10] is a new tool for aligning *in silico* contigs to a consensus optical map using an FM-Index. TWIN converts contigs into a restriction pattern by performing an *in silico* of the contig sequence. An FM-Index is constructed on the ordered integer sequence of restriction fragment lengths given by the consensus optical map, which allows for the efficient search for exact matches of patterns of *n* consecutive fragments. Once the FM-index is constructed, the run time is proportional to the number of fragments in the contig. To account for fragment sizing error, TWIN modifies the FM-Index backward search algorithm to backtrack along possible alignment choices that are consistent with the current fragment in the query. To reduce computational effort during the backtrack procedure, TWIN relies on an integer wavelet tree auxiliary data structure which allows the algorithm to focus on just those optical fragments within the current FM-index interval that are consistent with the current query fragment. A drawback of this algorithm is its inability to handle unmatched restriction sites such as those caused by missed fragments or restriction sites.

#### Significance of alignments

All alignment algorithms face the challenge that under any alignment scoring scheme, a given query restriction pattern may have multiple good quality alignments to the reference or consensus map. In cases when the alignment score depends on the number of restriction fragments and length of the query sequences, as in [7], a simple alignment score threshold is not sufficient to distinguish between ambiguous alignments. Sarkar *et al.* [11] observe that the optimal alignment scores of a query restriction pattern to permuted versions of the true reference map are highly correlated. In other words, the best alignment scores for spurious alignments depend on properties of the query map itself. The authors model the distribution of alignment scores for spurious alignments so they can use a map specific cutoff for determining alignment significance. In particular, the authors model the optical alignment score under the null hypothesis that the alignment is spurious using multiple linear regression on the number of query map fragments *N,* the map length *L*, and their product *NL*. The standard deviation of the optimal alignment score against a random spurious reference is modeled as a linear function of the mean optimal alignment score. The regression model is fit by aligning a set of query maps to a single permuted reference map, avoiding the computational bottleneck of performing a permutation test for each aligned query map against a set of permuted reference maps. Sarkar *et al.* also use logistic regression to predict the probability that a query map will have an alignment to a reference genome given the query map’s information content. This logistic model can be used to filter out query maps that are unlikely to align, saving computational resources. The authors demonstrate how an iterative optical map assembly algorithm performs better when using optical map alignments that are deemed significant using query-specific thresholds.

### Algorithms for optical map assembly

An optical mapping experiment produces a restriction map (Rmap) for a collection of DNA molecules on the order of ~500 kb in length. As in shotgun sequencing, these molecules are produced by randomly shearing the DNA from the organism of interest. It is therefore necessary to assemble the Rmaps in order to produce a more contiguous, higher quality consensus optical map. A consensus map is formed by computing a consensus restriction pattern for Rmaps that share compatible patterns and are therefore highly likely to have originated from the same place in the genome. Each assembled consensus restriction pattern is known as an optical map contig. Each optical map contig is characterized by both its consensus restriction pattern and a layout that provides the position and orientation of each Rmap used in its construction.

The Gentig algorithm [12] is the first published method for the assembly of consensus optical maps for shotgun optical mapping experiments. The method uses a Bayesian formulation, and seeks to maximize the *a posteriori* estimate of the consensus map assembled from the Rmaps. A prior probability distribution *H* on the consensus map is selected as a decreasing function of contig length, giving a prior bias for shorter (i.e., more assembled) contigs. This prior helps select assemblies that do a better job of overlapping and incorporating the experimental optical maps. Contigs are built by greedily merging the two best overlapping Rmaps or contigs, where overlaps are computed using dynamic programming. Overlaps are only considered if the match scores better than a specified threshold that controls for false overlaps between two unrelated restriction maps. Gentig constructs its prior and overlap scores using a probabilistic model which accounts for the errors inherent in optical mapping, including sizing errors, missing cut sites due to partial enzyme digestion, and false cut sites due to imaging artifacts.

While Gentig has successfully been used to assemble bacterial genomes, it does not scale well to larger genomes where the number of input Rmaps is large. Procedures have been developed to use Gentig in an iterative fashion for *de novo* optical map assembly of larger genomes by first randomly partitioning the input Rmaps into separate groups, and then running Gentig independently on the groups to produce a set of contigs. Since there may be duplicate or overlapping contigs between the independent assemblies, Gentig is used to assemble all of the contigs together to remove any redundancy, yielding a set of seed contigs. The input Rmaps are then aligned to the seed contigs as a means to cluster the Rmaps based on similarity, and then these “piles” of Rmaps are independently assembled using Gentig to produce a new set of contigs. This process is repeated for several iterations, producing a final set of contigs. Variations of this method have been used to build *de novo* optical map assemblies for *Leishmania major* Friedlin (34.7 Mb) [13], *Oryza sativa* (rice, 382 Mb) [14], *Zea mays L.* (maize, 2.5 Gb) [15], and *Melopsittacus undulatus,* (parakeet, 1.2 Gb) [16] *.*

Valoeuev *et al.* [8] have implemented an optical map assembler based on the overlap layout consensus (OLC) paradigm of sequence assembly. The overlap graph consists of Rmaps, represented as nodes, and significant overlaps, represented as edges between the Rmaps. First, pairwise overlaps are constructed between all of the Rmaps. This is the most computationally intensive step and is performed on a computing cluster. High scoring overlaps are selected to construct the overlap graph. The graph is cleaned by removing potential false overlaps by identifying paths through the overlap graph that are weakly supported. The set of edges is further refined by removing any edges which disagree with higher scoring information. Additional false edges are removed from the graph by considering edges that form a path between two nodes for which there is no alternative path with a consistent distance. Lastly, chimeric maps are identified as local articulation nodes. Valouev *et al.* demonstrate their optical map assembler by producing consensus maps for *Yersinia pestis* KIM, *Escherichia coli* K12, *Thalassiosira pseudonana*, *O. sativa* ssp *japonica* (rice), and *Homo sapiens*.

### Applications

#### Structural variation

A promising application of optical mapping technology is the characterization of structural variation within genomes. Optical mapping data span much longer genomic ranges that commonly achievable mate-pair sizes, and thus have the ability to detect large-scale variants that cannot be detected using paired end reads.

Teague *et al.* [4] have successfully used optical maps to detect structural variants in four normal human samples compared to the human reference genome, detecting both small variants, such as missing or extra enzyme cut sites, as well as large-scale insertions, deletions and inversions, ranging from thousands to millions of base pairs in size. Variants were detected by first constructing an optical consensus map for each sample using an iterative assembly strategy initially guided by an *in silico* map of the human reference. First, the Rmaps were aligned to the reference *in silico* map as a means to cluster the Rmaps with similar restriction patterns. Next, each cluster of maps was assembled using the Gentig software to produce a contig (i.e., consensus restriction pattern) for the cluster. The assembled contigs from all of the clusters were used in place of the reference in the second iteration, and the Rmaps were again aligned and assembled to produce a new set of Rmap contigs. This process was repeated for eight iterations, yielding a high quality consensus optical map for that sample. Structural variants between each assembled sample and the human reference were called by looking at the depth of Rmap coverage supporting each variant. A *p*-value was assigned to each variant call for missing cuts and extra cuts through a Binomial test and for indel calls using a Z-test derived from the sizing error model. The paper demonstrates that each of the four samples has hundreds of unique structural variants that are neither present in the other samples nor the human reference.

Optical mapping has also been used to characterize structural variants in oligodendroglioma [17], a type of brain cancer. A similar iterative assembly strategy was used to assemble a consensus optical map for two different tumor samples, HF087 and HF1551. Over 1,000 structural variants were called between each sample and human reference. In addition, a hidden Markov model (HMM) was trained on normalized Rmap coverage to determine the copy number at each chromosomal location. Loss of heterozygosity (LOH) events in which one copy of the chromosome is lost were observed in chromosomes 1, 14, 19, and 21. In addition, coverage analysis of Rmaps obtained from two adjacent slices of sample HF1551 revealed distinct LOH events for each slice, suggesting that these adjacent slices of the same tumor actually evolved from different cancer cell clones.

#### Genome assembly

Consensus optical maps provide long-range information over the length of a genome that can be used to aid in genome sequence assembly and validation. Assembly algorithms are graph based, where sequences are represented as nodes and overlaps between sequences are represented as edges. Each path through the assembly graph generates a sequence, and each possible path gives a possible reconstruction of the genome. Genomic repeats introduce nodes that must be traversed multiple times, thereby tangling the assembly graph.

AGORA [18] presents a method for guiding genome assembly to resolve repeats using optical maps by selecting the correct path among exponentially many paths consistent with the set of reads. AGORA works by first aligning long sequence contigs extracted from de Bruijn graph edges to the consensus optical map. All contigs with a unique placement give a genome wide scaffold (i.e., layout). Gaps in the scaffold are filled by greedily selecting a path in the de Bruijn graph between consecutively aligned contigs that is consistent with the restriction pattern of the optical map, thereby resolving repeats. The path is selected using a bounded depth-first search. Simulations with AGORA on error-free de Brujin graphs for bacterial genomes and simulated optical maps suggest that high quality consensus optical maps can accurately improve assembly contiguity.

Xavier *et al.* [19] have demonstrated how optical consensus maps can be used to assess assembly accuracy when selecting from a set of candidate assemblies constructed under different assembly parameter settings. In a de Bruijn graph assembly, a critical parameter is the *k-mer* length, which controls the length of the overlap used. Generally, a larger *k-mer* setting results in a more aggressive assembly that improves assembly contiguity at the expense of accuracy, while a smaller *k-mer* setting gives a conservative but accurate assembly at the expense of contiguity, as the de Bruijn graph has branches for genomic repeats of length ≥ *k*. Xavier *et al.* built multiple *de novo* assemblies for Methicillin-resistant *Staphylococcus aureus* (MRSA) using different assemblers and a wide range of *k-mer* settings. The authors detected mis-assemblies by finding contigs that have a split alignment to the optical consensus map, then selected the assemblies with highest contiguity (i.e., with the most resolved repeats), but which did not exhibit any mis-assemblies with respect to the optical map.

Furthermore, optical maps have also proven useful for validating existing genome assemblies and characterizing mis-assemblies. In the case of the *O. sativa* (rice) genome [14], an optical consensus map was used to compare the quality of two independently constructed assemblies, one by TIGR and the other by the International Rice Genome Sequencing Project. Consensus optical maps have also been used as part of the Assemblathon 2 competition [20] to assess the quality of *de novo* assemblies for a budgerigar (*Melopsittacus undulatus*) a Lake Malawi cichlid (*Maylandia zebra*), and boa constrictor (*Boa constrictor constrictor*). The consensus optical maps were iteratively assembled using Gentig. Assembly quality was assessed by aligning sequence scaffolds constructed from paired-end reads to the optical consensus map under different levels of alignment stringency. Scaffolds that globally align to the optical map under the most restrictive setting are considered correct, while scaffolds that only have local alignments are considered to have mis-assemblies.

## Conclusions

In this paper we have reviewed algorithms and tools for processing optical mapping data (alignment and assembly) and for using these data to identify structural variants, and to guide or validate genome assemblies. Due to the long range information provided by optical mapping data (potentially spanning hundreds of kilo-base-pairs or more) and the relatively complex and error-prone approaches for constructing long mate-pair libraries in the context of modern sequencing technologies, optical mapping data hold tremendous promise in supplementing or even replacing sequencing data in the study of chromosomal rearrangements.

Despite this promise, as you can see from our review, relatively few methods exist for analyzing and using optical mapping data, and even fewer are available in effective publicly-available software packages. While Gentig has successfully been used to assemble consensus optical maps for bacterial genomes, it does not scale well to large genomes, and the software is not freely available. Beyond AGORA, which is a proof of concept implementation, no genome assembler can make use of optical mapping information. Furthermore, there are virtually no tools available for using optical maps to characterize structural variants. The alignment tools reviewed above could and have been used for this purpose, but only through the manual curation of the raw alignment output rather than through the use of specialized structural variant discovery tools. There is, thus, a critical need for the continued development and public release of software tools for processing optical mapping data, mirroring the tremendous advances made in analytical methods for second- and third-generation sequencing data.

## Abbreviations

HMM: Hidden Markov model
LOH: Loss of heterozygosity
MRSA: Methicillin-resistant *Staphylococcus aureus*
OLC: Overlap layout consensus.
